# Circulating tumor-derived mutant mitochondrial DNA: a predictive biomarker of clinical prognosis in human squamous cell carcinoma

**DOI:** 10.18632/oncotarget.523

**Published:** 2012-07-25

**Authors:** Katsuhiro Uzawa, Takao Baba, Fumihiko Uchida, Masanobu Yamatoji, Atsushi Kasamatsu, Yosuke Sakamoto, Katsunori Ogawara, Masashi Shiiba, Hiroki Bukawa, Hideki Tanzawa

**Affiliations:** ^1^ Department of Clinical Molecular Biology, Graduate School of Medicine, Chiba University, Chiba, Japan; ^2^ Department of Dentistry-Oral and Maxillofacial Surgery, Chiba University Hospital, Chiba, Japan; ^3^ Department of Oral and Maxillofacial Surgery, Clinical Sciences, Graduate School of Comprehensive Human Sciences, University of Tsukuba, Ibaraki, Japan

**Keywords:** squamous cell carcinoma, mitochondrial DNA, high-resolution melting curve analysis, circulating tumor-derived DNA, prognosis

## Abstract

While circulating tumor-derived molecules have been identified in a variety of malignant tumors, it is sometimes difficult to detect the molecular targets due to the lower serum concentration. We report that evaluation of circulating tumor-derived mitochondrial DNA (mtDNA) seems to have novel efficiency for detecting tumoral micrometastasis. In murine xenografting human oral cancer cells, human mtDNAs could be quantitatively detected from multiple organs and blood samples whereas human nucleic DNAs could not. We also determined if this mtDNA blood test was relevant for patients with oral cancer with no histologic evidence of tumoral cells in their surgical margins. For this, mtDNA from normal and tumorous tissues and serum mtDNA obtained pre and postoperatively was examined at three different regions including the displacement loop, 12S-rRNA, and 16S-rRNA. All recurring patients had significantly higher amounts of mutant mtDNAs in the tumoral tissues compared with the non-recurring group. More importantly, on the blood test with the cut-off point by receiver operating characteristic (ROC) curve analysis, while the vast majority of serum mtDNA samples obtained postoperatively in the recurring cases maintained significantly higher amounts of mutant mtDNAs, the non-recurring cases did not, and they showed good prognosis. This is the first report of this approach for managing patients after resection of oral tumors, and may have substantial diagnostic potential for other tumoral types.

## INTRODUCTION

Although the status of surgical margins and/or regional lymph node metastasis are relevant prognostic factors in malignant tumors including oral squamous cell carcinoma (OSCC), local recurrences and/or distant metastasis (r/m) or both have developed in a subset of patients with OSCC with histologically negative margins and lymph nodes. Despite its clinical importance, there is no relative blood test for early detection of tumoral recurrences and regional/distant metastases.

It has been well documented that detection of circulating tumor-associated molecules, such as mutant genomic DNA (gDNA), methylated DNA, and tumor-specific mRNA/micro-RNA could be useful for predicting a variety of recurrences of tumors in humans or metastases [1-7]. Microsatellite alterations were first identified in serum DNA of patients with advanced regional/distant metastases in head and neck SCCs (HNSCCs) including OSCCs [8]. We reported that monitoring circulating tumor-associated DNA by microsatellite analysis at nine chromosomal loci could be detected and also might be an early diagnostic tool for use postoperatively in patients with OSCC [9]. Currently, screening of tumor-associated molecules in HNSCCs has primarily focused on qualitative examination of genetic abbreviations. In addition, from a clinical standpoint, the detection of mutant nuclear genes sometimes can be limited due to the low plasma concentration. Some patients with recurrent HNSCC but not those without recurrences had mitochondrial DNA (mtDNA) mutations in their histologically negative margins, suggesting the potential usefulness for monitoring patients [10].

Based on this, the current study sought to determine if mutant mtDNA, which has much higher cellular copy numbers than those of genomic DNA (gDNA), can be detected quantitatively in the sera of patients with OSCC, and if so, whether that detection reflects the diagnostic relevance of the method for early disease detection and prognosis. Cases were selected for retrospective analysis that had a recurrence or metastasis postoperatively to evaluate whether tumor-associated mutant mtDNA detection in the sera, diagnosed as tumor-free by conventional histologic analyses, could be a novel prognostic biomarker.

## RESULTS AND DISCUSSION

To identify the sequence variation of the human mtDNA genome in primary cultured human normal oral keratinocytes (hNOKs) and OSCC cells (Sa3 and HSC-4), 21 sets of PCR primers were designed to cover the entire regions at the displacement (D)-loop, 12S-rRNA, and 16S-rRNA of the mtDNA genome (Supplementary Table 1), where frequent mutations have been identified in various cancer types [11-15]. The results of DNA sequence analysis were compared with the MITOMAP database (www.mitomap.org/MITOMAP/Human MitoSeq). We detected three novel somatic mutations in the regions of D-loop (C:G to T:A at position 68), 12S-rRNA (A:T to G:C at position 1107) in Sa3 cell line, and 16S-rRNA (T:A to C:G at position 3190) in the HSC-4 cell line, whereas the mtDNA sequences from the hNOKs completely matched the MITOMAP database.

**Figure 1:**
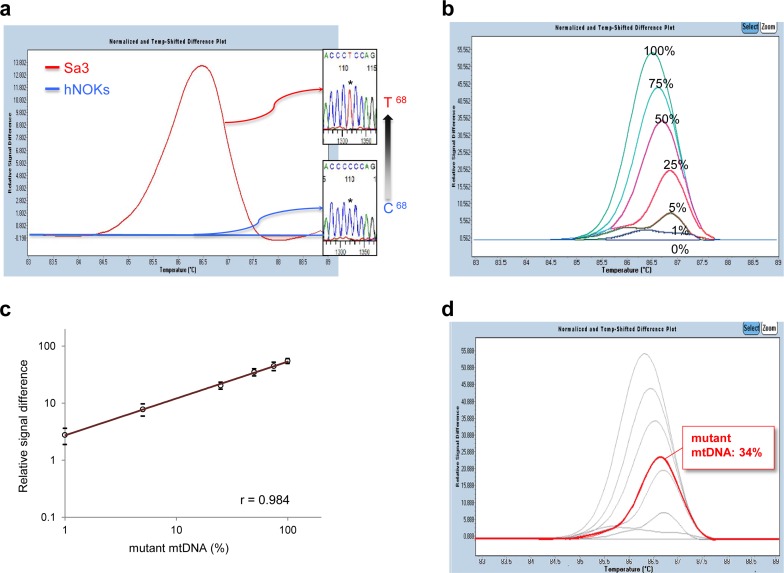
Mutational analyses of human mtDNA including the regions of the D-loop, 12S-rRNA, and 16S-rRNA in human OSCC-derived cells, Sa3 and HSC-4 (A) A typical qPCR-HRMA result followed by DNA sequence analysis of Sa3 cells clearly shows a distinguishable peak (red line) as a result of C68T in the D-loop region. Primary cultured hNOKs, indicating the baseline level (blue line) in the qPCR-HRMA panel, were obtained from healthy donors and used as a normal control. (B) A representative result of the standard curve for the D-loop region in the mtDNA genome. The standard curves were created by diluting mutated mtDNAs (D-loop-C68T) with wild-type mtDNAs to prepare 100%, 75%, 50%, 25%, 5%, 1%, and 0% mutated samples for detecting the D-loop region in the mtDNA genome. (C) A plotted standard curve for the D-loop region. The levels of the relative signal differences obtained by the qPCR-HRMA (y-axis) are reported as percentages of the mutant mtDNA. The coefficient of correlation (r = 0.984) is high. (D) Determination of the mutant mtDNA level in the serum from a Sa3-xenografted mouse (mouse 1). The fluorescence of the serum sample (red line) normalized as a differential signal against each standard curve in light grey, enabling detection of 34% mutant mtDNA in the D-loop region.

To detect the mtDNA mutations sensitively, easily, and rapidly, we then used quantitative real-time PCR combined with high-resolution melting curve analysis (qPCR-HRMA) [16]. HRMA is a rapid, specific, and cost-effective method for detecting specific nucleotide changes not only for genotyping single-nucleotide polymorphisms but also specific gene mutations in a variety of malignant tumors [17-20]. However, the sensitivity and specificity of HRMA depend on the amplicon length of the PCR products [21,22]. Thus, to determine the optimal condition of the qPCR-HRMA, three sets of PCR primers were prepared for the recommended amplicon length range (<300 basepairs) [21] in the specific region that includes each mtDNA mutation identified. On qPCR-HRMA, melting curves that correlated with the mutations in the mtDNA genome of the cell lines examined were clearly separated on the HRMA chromatogram (Figure 1a). HRMA could be carried out quantitatively based on the standard curve generated by serial dilutions of the control DNA [23]. To quantify the amount of mutant mtDNA in blood and tissue samples, we diluted mutated mtDNAs (D-loop-C68T, 12S-A1107G, and 16S-T3190C) with wild-type mtDNAs to prepare 100%, 75%, 50%, 25%, 5%, 1%, and 0% of mutated samples and created a standard curve (Figure 1b). The percentage of mutated mtDNA was determined with reference to the external standard curve. We also generated typical standard curves by calibrating the values of differential fluorescence peaks for each dilution and obtained relatively good calibration of the percentages of mutant mtDNA (Figure 1c). A control experiment with the qPCR-HRMA combined with the standard curve was performed to determine whether human oral cancer cells could be detected quantitatively from peripheral blood samples and multiple organs in a human OSCC cell line (Sa3) from xenografted nude mice. Although no visible metastatic lesions were observed in distant organs, such as the lung, liver, kidney, and spleen, the Sa3 specific mutant mtDNA sequences, which are C:G to T:A at position 68 and/or T:A to C:G at position 3190 in the mtDNA genome, were detected consistently in the organs of the mice injected with the Sa3 cells (with the exception of the lung from one mouse) but not in those of control mice (Supplementary Table 2). Further, it is noteworthy that mutated mtDNA was identified in the serum from all the Sa3-xenografted mice (Supplementary Table 2). Figure 1d shows a typical result for mouse 1 whose serum sample contained 34% of mutant mtDNA in the D-loop region based on the standard curve. These observations indicated that this approach seems suitable for rapid and efficient measurement for detecting mutant mtDNAs and has a potential advantage for clinical use.

To verify that this relatively high level of sensitivity also was useful in patients with oral cancer, we examined primary OSCCs and matched both corresponding normal oral epithelial tissues and sera from 61 individuals. Supplementary Table 3 shows the outline of the search flow diagram between the r/m− and r/m+ cases. Blood samples were collected from patients preoperatively and 4 weeks postoperatively, and of those, 45 were diagnosed postoperatively as having a good prognosis (r/m−), and 16 had a local recurrence and/or distant metastasis within 9 months postoperatively (r/m+). Overall, 244 clinical samples obtained from patients with OSCC were analyzed using qPCR-HRMA. We compared the levels of mutant mtDNA between tumoral tissues and matched normal counterparts based on the standard curve created. As expected, mutant mtDNA was identified in samples from tumoral tissues, whereas a relatively low level of the mtDNAs was observed in normal tissues in any regions examined (*P*<0.01 at the D-loop and 16S-rRNA; *P*<0.05 at 12S-rRNA) (Supplementary Figure 1). When we compared the mtDNA levels in tumoral tissues between the r/m+ group and r/m− group, the difference was significant (*P*<0.05, Supplementary Figure 2). The current results suggested that assessment of mutant mtDNA in tumoral tissues might be a potential prognostic predictor for patients with OSCC.

**Figure 2:**
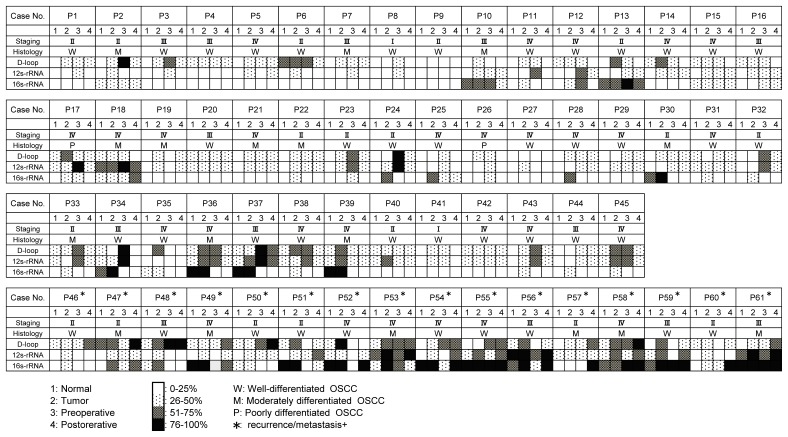
Status of mutant mtDNA from 61 patients with OSCC with surgical malignancy-free margins Note that all patients with a good prognosis, except for three patients 13, 18, and 37, had less than 50% of mutant mtDNA in their sera postoperatively. In contrast, all r/m+ patients with no exceptions had more than 50% of mutant mtDNA in at least one region examined. P = patient.

**Figure 3:**
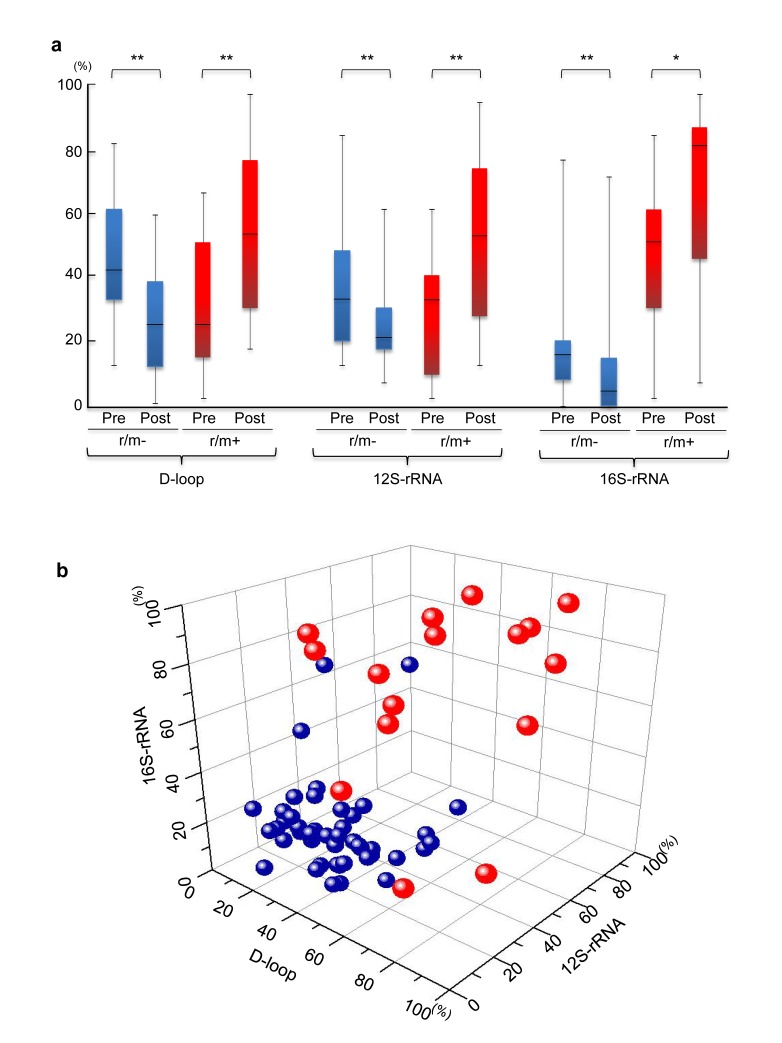
(A) The status of mutant mtDNA levels in patients with OSCC with (red boxes) or without (blue boxes) r/m preoperatively or postoperatively The r/m− groups have significantly decreased mutant mtDNAs postoperatively compared to preoperatively, whereas significantly increasing mutant mtDNA levels were detected at all regions in sera obtained postoperatively from r/m+ patients. The statistical significance of the data was determined using the Mann-Whitney *U* test. *P*<0.05 was considered significant. *P<0.05; **<0.01. The data are expressed as the mean ± standard error of the mean. The horizontal lines indicate the medians. All experiments were performed in triplicate. (B) To more clearly illustrate the specificity of the status of mutant mtDNA levels in the three different regions, we used Origin 8.6 software (OriginLab Co., Northampton, MA, USA) to create a three-dimensional scatterplot with an x-axis indicating the levels in the D-loop, a y-axis for the 12S-rRNA, and a z-axis for the 16S-rRNA, respectively. Data point sets from 61 patients with OSCC are plotted as circles. Most of the r/m− patients (blue) are very close (below 50%) on the X (D-loop)-Y (12S-rRNA)-Z (16S-rRNA) plane; the r/m+ group tends to indicate higher (>50%) amounts of mutant mtDNA.

We then determined whether the relatively high sensitivity of the qPCR-HRMA could be used for a clinical blood test. First, we preliminarily compared the levels of gDNA and mtDNA in the sera of 61 patients with OSCC. As expected, the level of mtDNA was significantly (*P*<0.05) higher than that of gDNA (3576.5 ± 710.9 ng/μl for mtDNA vs. 17.0 ± 6.8 ng/μl for gDNA). In addition, mtDNA in the sera, only 50 to 100 μl was needed for the analysis, whereas more than 1,000 μl of serum was needed to detect microsatellite alterations in gDNA [9]. Much smaller amounts of sera were required from patients with colorectal cancer for mutant mtDNA evaluation compared to those for detecting mutant gDNA [24]. Although we did not find a significant difference in the amount of serum mtDNA between the r/m+ group and r/m− group (data not shown), the previously mentioned data indicated that circulating mtDNA detection rather than gDNA detection might be a potential valuable blood test for use in patients with cancer. To test this idea, we studied the serum of 61 patients before and after surgery to remove their tumors. All patients except patient 9 had mutant mtDNA molecules in at least one mtDNA template molecule from the resected tumors and the sera before and after surgery (Figure 2). The patients with recurrences or metastases postoperatively had much higher concentrations of mutant mtDNA in the sera 4 weeks postoperatively in any target region examined (Figure 3a, b), even if the patients were diagnosed histologically as tumor free at their surgical margins. In general, the OSCC cases indicated that a more differentiated phenotype resulted in a better prognosis. However, there was no correlation between tumoral differentiation and mutant mtDNA detection in the sera from the group with a poor prognosis. We have no exact explanation for why these patients had mutant mtDNAs even at the last point examined. The proposed mechanisms might include differences in tumor cell activation and numbers, the presence of nonneoplastic cells with accumulating genetic changes in the regions surrounding the site of the tumor, and deteriorated autoimmune systems.

**Figure 4:**
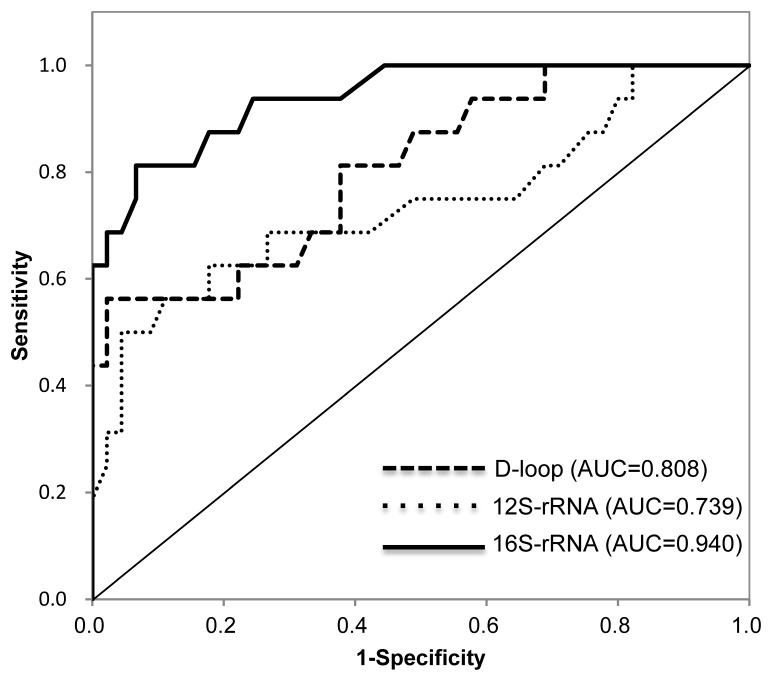
ROC curves and AUC for the D-loop, 12S-rRNA, and 16S-rRNA To evaluate the diagnostic relevance for predicting the r/m of serum mutant mtDNAs, we used the ROC curve by plotting sensitivity versus specificity. The AUCs for mutant mtDNAs are 0.808 for the D-loop, 0.739 for the 12S-rRNA, and 0.940 for the 16S-rRNA, indicating that the diagnostic potential of the level of serum mutant mtDNA 4 weeks after surgery is confirmed as indicated by the AUC values for the different regions examined. The thinner solid line indicates the diagonal representing a hypothetical test with no diagnostic discrimination. The statistical significance of the study data was determined using the Mann-Whitney *U* test. *P*<0.05 was considered significant. Data are expressed as the mean ± standard error of the mean. Statistical analyses were performed using SPSS 17.0 software (SPSS Inc., Chicago, IL, USA).

Since we could not detect a significant difference between the presence of r/m+ and the conventional blood test for SCC antigen levels in the sera (Supplementary Table 2), the diagnostic accuracy of the identified biomarkers was assessed using ROC curve analysis (Figure 4). The optimal threshold value was 52% for D-loop (sensitivity, 56.3%; specificity, 97.8%), 56% for 12S-rRNA (sensitivity, 56.0%; specificity, 95.6%), and 47% for 16S-rRNA (sensitivity, 81.3%; specificity, 93.4%), respectively. When the cutoff values for the mutant mtDNA levels were set at 52% for the D-loop, 56% for the 12S-rRNA, and 47% for the 16S-rRNA, each area under the ROC curve (AUC) for the mtDNA was 0.808 (95% confidence interval [CI], 0.680-0.935, *P*<0.001) for the D-loop, 0.739 (95% CI, 0.578-0.900, *P*<0.01) for the 12S-rRNA, and 0.94 (95% CI, 0.877-0.999, *P*<0.001) for the 16S-rRNA (Figure 4), suggesting that all mtDNA targets can be independent prognostic markers for evaluating high-risk groups of patients with OSCC postoperatively. In addition, even if we analyzed for each single mtDNA target, there was still a significant correlation to distinguish OSCC from the r/m+ and r/m− groups. These data supported the concept that measuring the circulating serum mtDNA could be an appropriate strategy for differential diagnosis in high-risk groups of patients with OSCC postoperatively.

Although the current study included a small number of clinical samples with a limited target region in the mitochondrial genome, our results indicated a potential use for clinical diagnostic application. We believe that by increasing the number of samples with high sensitivity mutation detection spanning the entire mitochondrial genome (e.g., MitoChip array technique), the diagnostic model and increased accuracy of the diagnostic predictions can be refined.

## MATERILS AND METHODS

### Ethics Statement

This study protocol was approved by the Ethical Committee of Graduate School of Medicine, Chiba University (No. 236) and was performed in accordance with the ethical standards laid down in the Declaration of Helsinki. All patients provided informed consent for the study protocol.

### Mitochondrial DNA sequencing for human OSCC derived cell lines

Sa3 cell line was purchased from the RIKEN Bio-Resource Center through the National Bio-Resource Project of the Ministry of Education, Culture, Sports, Science and Technology, Tsukuba, Japan, and HSC-4 was purchased from the Human Science Research Resources Bank (Osaka, Japan), which conducted cell lines authentication by DNA barcoding. They were grown in Dulbecco's modified Eagle medium/F-12 HAM (Sigma-Aldrich Co, St. Louis, MO) supplemented with 10% fetal bovine serum (Sigma) and 50 units/ml penicillin and streptomycin (Sigma).

To avoid gDNA contamination for mitochondrial DNA extraction in the present study, we used the QIAamp UltraSens Virus Kit (QIAGEN) and mtDNA Extractor CT Kit (Wako, Osaka, Japan) according to the manufacturer's instructions. To search for state of mitochondrial DNA mutation, the entire coding sequences of the three regions including D-loop, 12S-rRNA, and 16S-rRNAwere examined with 21 sets of PCR primers (Supplementary Table 1). All PCR products were subjected to electrophoresis through a 1.5% agarose gel and stained with ethidium bromide. The target DNA bands were extracted using the MinElute Gel Extraction Kit (QIAGEN, Valencia, CA, USA). Each PCR product was subcloned into a pCR8/GW/TOPO TA cloning vector (Invitrogen, Carlsbad, CA, USA) and sequenced. The results of DNA sequence analysis were compared with the MITOMAP database (www.mitomap.org/MITOMAP/Human MitoSeq).

### qPCR-HRMA

We optimized the conditions and examined the feasibility of using qPCR-HRMA for detecting three different mutations (D-loop-C68T, 12S-A1107G, and 16S-T3190C). The following PCR primers were designed and used for the analyses. The primer sequences were: D-loop region: Forward 5'-GCTCTCCATGCATTTGGT-3' (38-55), Reverse, 5'-ACACTTTAGTAAGTATGTTCGCC-3' (206-184); 12S-rRNA region: Forward, 5'-ATATCTGAACACACAATAGCTAAGAC-3' (1039-1064), Reverse, 5'-TAGAGGGATATGAAGCACCG-3' (1196-1177); and 16S-rRNA region: Forward, 5'-GTAATCCAGGTCGGTTTCTA-3' (3082-3101), Reverse, 5'-ACCGGGCTCTGCCATCTTAA-3' (3250-3231). Using these PCR primer sets, qPCR-HRMA was performed using a LightCycler 480 system (Roche Diagnostics GmbH, Mannheim, Germany) in a final volume of 20 μl of a reaction mixture comprised of 10 μl of LightCycler 480 High Resolution Melting Master mix (Roche), 3 mM of MgCl_2_, and 4 μM of the primers, according to the manufacturer's instructions.

The standard curves were created by each diluting mutated mtDNA from Sa3 or HSC-4 with wild-type mtDNAs to prepare 100%, 75%, 50%, 25%, 5%, 1%, and 0% mutated samples for detecting the D-loop region in the mtDNA genome.

To test whether human oral cancer cells could be detected quantitatively from peripheral blood samples and multiple organs, we transplanted Sa3 cells (2 × 10^6^) subcutaneously injected into the BALB/cAnNcrj-nu/nu mice (n=2, Charles River Japan), which were sacrificed after 6 weeks. The non Sa3 transplanted mice (n=2) were used as control. We then excised several organs including the tumor, lungs, liver, kidney and spleen. Serum samples were also collected from them. Mitochondrial DNA was extracted from them for qPCR-HRMA. All mice were maintained under specific pathogen-free conditions. All animal experiments were conducted in accordance with the guidelines of the Chiba University laboratory animal center.

### Determination of mutational mitochondrial DNAs in OSCC patients

Sixty-one patients with OSCC with surgical malignancy-free margins were selected for this study. No patients underwent a blood transfusion. Patients with no autoimmune disease were selected. Forty-five were diagnosed postoperatively as having a good prognosis (r/m−) and 16 had a local recurrence and/or distant metastasis within 9 months postoperatively (r/m+). Detailed patient information is shown in Supplementary Table 3. Overall, 244 mtDNA samples were collected from surgical tissues including corresponding normal oral epithelium and tumor and also sera from preoperatively and postoperatively, and they were stored at −80°C until use. The qPCR-HRMA for the regions at D-loop, 12S-rRNA and 16S-rRNA was performed as the same manner as described above.

### Statistical analyses

All experiments were performed in triplicate. The statistical significance of the data was determined using the Mann-Whitney *U* test. *P*<0.05 was considered significant. * indicates *P*<0.05 and ** indicates *P*<0.01. The data are expressed as the mean ± standard error of the mean. The three-dimensional scatterplot with an x-axis indicating the levels in the D-loop, a y-axis for the 12S-rRNA, and a z-axis for the 16S-rRNA was created by the Origin 8.6 software (OriginLab Co., Northampton, MA, USA). To evaluate the diagnostic relevance for predicting the r/m of serum mutant mtDNAs, we used the receiver operating characteristic (ROC) curve analysis by plotting sensitivity versus specificity, and area under the ROC curve (AUC) values with estimate odds ratios and 95% confidence intervals (CIs). Data are expressed as the mean ± standard error of the mean. Statistical analyses were performed using SPSS 17.0 software (SPSS Inc., Chicago, IL, USA).

## Supplementary Tables and Figures




