# Supplementary material for: Circulating tumor-derived mutant mitochondrial DNA: a predictive biomarker of clinical prognosis in human squamous cell carcinoma
Source: Oncotarget. 2012 Jul 25;3(7):670–7. doi: 10.18632/oncotarget.523 (PMC3443250; doi:10.18632/oncotarget.523)
Supplement: Supplementary file 1 [file oncotarget-03-670-s001.docx]

**Circulating tumor-derived mutant mitochondrial DNA: a predictive biomarker of clinical prognosis in human squamous cell carcinoma - Katsuhiro Uzawa et al**

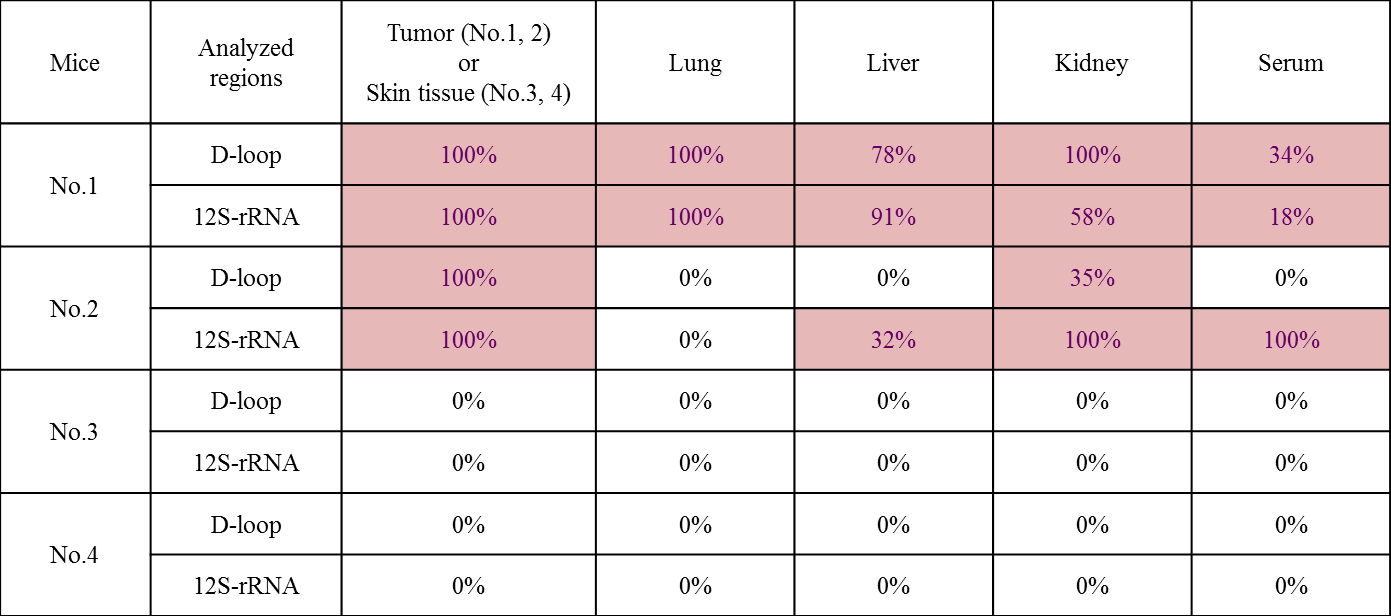


Supplementary Table 2: Comparison of Sa3 specific mutant mtDNA levels between xenografted and control mice
