# Supplementary material for: Circulating tumor-derived mutant mitochondrial DNA: a predictive biomarker of clinical prognosis in human squamous cell carcinoma
Source: Oncotarget. 2012 Jul 25;3(7):670–7. doi: 10.18632/oncotarget.523 (PMC3443250; doi:10.18632/oncotarget.523)
Supplement: Supplementary file 2 [file oncotarget-03-670-s002.pdf]

Circulating tumor-derived mutant mitochondrial DNA: a predictive biomarker of clinical prognosis in human squamous cell carcinoma - Katsuhiro Uzawa et al

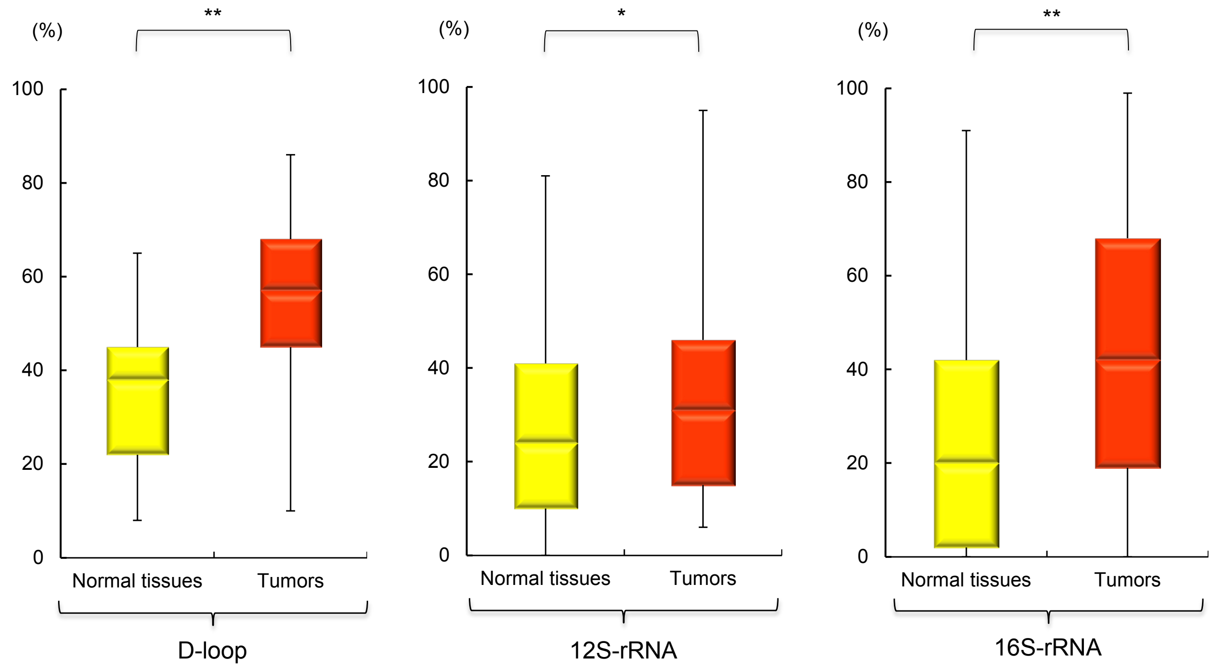

Supplementary Figure 1: Comparison of mutant mtDNA levels between tumors (red boxes) and corresponding normal tissues (yellow boxes). The statistical significance of the data was determined using the Mann-Whitney *U* test. $P<0.05$ was considered significant. The data are expressed as the mean ± standard error of the mean. The horizontal indentations in the colored boxes indicate the medians. *P<0.05, **P<0.01 compared with normal tissues. All experiments were performed in triplicate.

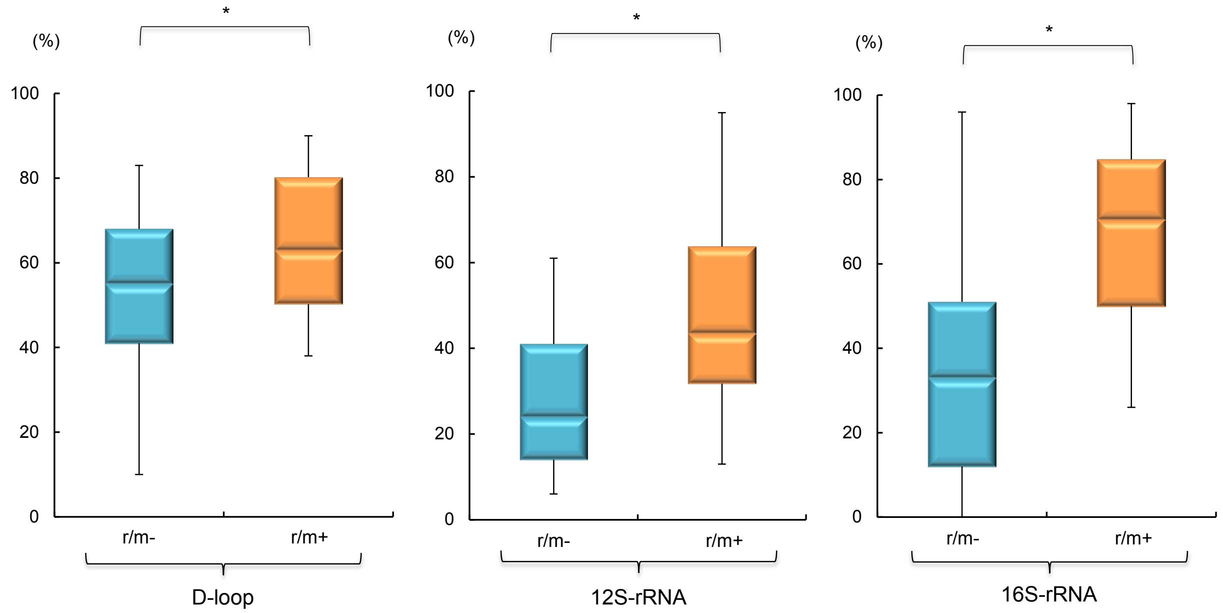

Supplementary Figure 2: Comparison of the levels of mutant mtDNAs in the tumors obtained from r/m- and rm+ patients. The blue boxes indicate the r/m- groups; the orange boxes indicate the r/m+ groups. The statistical significance of the data was determined using the Mann-Whitney *U* test. $P<0.05$ was considered significant. The data are expressed as the mean ± standard error of the mean. The horizontal indentations in the colored boxes indicate the medians. *P<0.05 compared with the r/m- group. All experiments were performed in triplicate.
